# The Role of Microbiota in the Immunopathogenesis of Endometrial Cancer

**DOI:** 10.3390/ijms23105756

**Published:** 2022-05-20

**Authors:** Małgorzata Sobstyl, Peet Brecht, Anna Sobstyl, Paulina Mertowska, Ewelina Grywalska

**Affiliations:** 1 Department of Gynecology and Gynecological Endocrinology, Medical University of Lublin, 20-037 Lublin, Poland; malgorzata.sobstyl@umlub.pl; 2 Department of Experimental Immunology, Medical University of Lublin, Chodźki 4a St., 20-093 Lublin, Poland; 54591@365.uml.edu.pl (P.B.); 53370@student.umlub.pl (A.S.)

**Keywords:** dysbiosis, endometrial cancer, endometrial microbiome, estrobolome, estrogen metabolism, inflammation

## Abstract

The female reproductive tract hosts a specific microbiome, which plays a crucial role in sustaining equilibrium and good health. In the majority of reproductive women, the microbiota (all bacteria, viruses, fungi, and other single-celled organisms within the human body) of the vaginal and cervical microenvironment are dominated by *Lactobacillus* species, which benefit the host through symbiotic relationships, in comparison to the uterus, fallopian tubes, and ovaries, which may contain a low-biomass microbiome with a diverse mixture of microorganisms. Although disruption to the balance of the microbiota develops, the altered immune and metabolic signaling may cause an impact on diseases such as cancer. These pathophysiological modifications in the gut–uterus axis may spark gynecological cancers. New information displays that gynecological and gastrointestinal tract dysbiosis (disruption of the microbiota homeostasis) can play an active role in the advancement and metastasis of gynecological neoplasms, such as cervical, endometrial, and ovarian cancers. Understanding the relationship between microbiota and endometrial cancer is critical for prognosis, diagnosis, prevention, and the development of innovative treatments. Identifying a specific microbiome may become an effective method for characterization of the specific microbiota involved in endometrial carcinogenesis. The aim of this study was to summarize the current state of knowledge that describes the correlation of microbiota with endometrial cancer with regard to the formation of immunological pathologies.

## 1. Introduction

**Figure 1:**
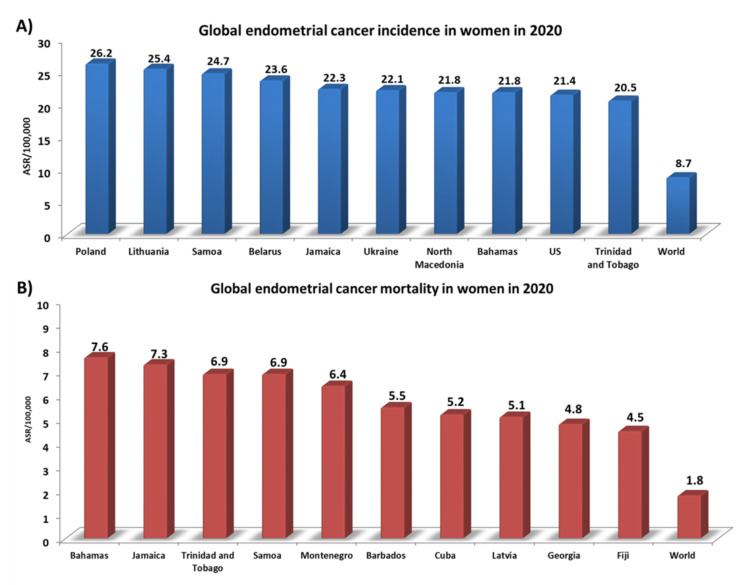
Statistics on the incidence and number of deaths from endometrial cancer in 2020. (**A**) Global endometrial cancer incidence in women in 2020; (**B**) Global endometrial cancer mortality in women in 2020. Abbreviations: ASR: age-standardized rate (based on [2]).

Endometrial cancer is the 6th most common cancer in women and the 15th most common cancer worldwide [1]. Over 417,000 new cases of endometrial cancer were reported in 2020, according to data presented by the World Cancer Research Fund International. Detailed analyses have shown that the incidence rate for this type of cancer worldwide is 8.7 ASR per 100,000 people [2]. However, 10 countries significantly exceed this level: Poland (26.2), Lithuania (25.4), Samoa (24.7), Belarus (23.6), Jamaica (22.3), Ukraine (22.1), North Macedonia (21.8), the Bahamas (21.8), the USA (21.4), and Trinidad and Tobago (20.5) (Figure 1A). In addition, the highest number of deaths from endometrial cancer in 2020 (which globally was 1.8 ASR per 100,000) was analyzed, with the ranking being topped by the Bahamas (7.6), Jamaica (7.3), and Trinidad and Tobago (6.9) (Figure 1B) [2]. Several non-genetic risk factors for endometrial cancer are well established, such as exogenous or endogenous estrogens caused by obesity [3], early age at menarche, late menopause [4], the use of tamoxifen, advanced age [5], diabetes mellitus [6], hypertension, and Lynch syndrome [7]. There are reports in the literature that some ovarian cancer histotypes (such as ovarian clear-cell carcinoma (OCCC) or endometriotic carcinoma (EOC)) may result from the development of endometriosis. This mainly concerns changes at the level of molecular pathology due to the presence of common mutations in cancer-related genes [8]. However, a study conducted by Pool et al. in 2017 on a prospective cohort of American nurses found that women diagnosed with endometriosis were not at a higher risk of developing endometrial cancer, unlike the well-described relationship between endometriosis and the development of ovarian cancer [9]. This is also confirmed by the present literature, which does not confirm an increased risk of neoplasms other than EOC in women with endometriosis [10]. Despite this, only half of the women diagnosed with endometrial cancer have common risk factors. The remaining other half are healthy-weight individuals who have never been treated with exogenous estrogens; they account for 20–25% of perimenopausal women, and 5% are under the age of 40 [11]. Recent evidence suggests that the microbiome may also be a risk factor. The human microbiota, which consists of diverse microbial taxa that coexist in the human body, reaches up to 100 trillion microbes [12]. These microbes include bacteria, viruses, yeasts, phages, and fungi, and are found in distinct ecological niches of the human body, such as the mouth, skin, lungs, gut, and genitourinary area. Microorganisms play a vital role in the development of up to 15% of cancers [13]. So far, there is significant evidence of microbial influence on the etiology and progression of gastric cancer and *Helicobacter pylori* [14]. Recent high-throughput sequencing (metataxonomic method) research has already been well established for colorectal cancer and infection with *Fusobacterium* [15] and *Porphyromonas* [16,17] which, furthermore, might be involved in a broader microbiome role in cancerous processes.

Moreover, dysfunction of the microbiome–brain–gut axis has been implicated in many neurological and psychiatric diseases, such as depression and Alzheimer’s disease [18], and may lead to several immune-related diseases, such as allergic rhino conjunctivitis, atopic eczema, or asthma [19].

Until recently, the most widely accepted explanation based on the work of Henry Tissier in 1900 assumed that a healthy uterine cavity is sterile. This statement was putative due to the barrier of bacterial ascension from the vagina caused by the cervical plug [20]. The early misconception about the sterility of the upper reproductive tract was subsequently challenged by multiple reports that proved the existence of uterine-dwelling bacteria in women [21,22]. Additionally, it has been demonstrated that the cervical mucus plug is not completely resistant to the ascension of bacteria from the vaginal microbiota [23]. To date, it has also been proven that the naturally occurring uterine peristaltic pump aids in the transport of sperm from the cervical canal to the uterus, and these peristaltic contractions have been shown to transport microspheres from the canal into the uterus and other areas of the upper female reproductive tract, suggesting that they may contribute to seeding the uterus with bacteria [24,25]. It has been demonstrated that the follicular phase of the menstrual cycle is connected with an increased frequency of uterine contractions [26]. Additionally, uterine abnormalities such as hyper- and dysregulation of uterine contractions may facilitate bacterial seeding of the uterus [27].

It has been suggested that an imbalance or dysbiosis of microbial populations along the female reproductive tract may be implicated in the pathology of gynecological cancer [27]. The uterus is a rare tissue type. According to estimates, the uterine microbiome contains between 100 and 10,000 times fewer microorganisms compared to the vaginal microbiome [28,29]. Since the uterus is a low-abundance site, more advanced methods for measuring bacterial levels are required. This became possible with the adoption of next-generation sequencing (NGS) technology in 2007, which enables a much broader assessment of the uterine bacterial composition, unlike previous achievements using culture-dependent approaches.

Taking the above into consideration, it can be stated that the endometrial microbiota’s diversity and composition play a pivotal role in relation to the immunopathogenesis of endometrial cancer. The aim of this study was to summarize the current state of knowledge by stating that dysbiosis of the microbiome is associated with a variety of pathologies, including female reproductive tract malignancies, a detailed characterization of species variation, and host–microbe interaction, which may provide clues for the prognosis, prevention, and identification of early diagnostic hallmarks, leading towards new therapeutic interventions.

**Figure 2:**
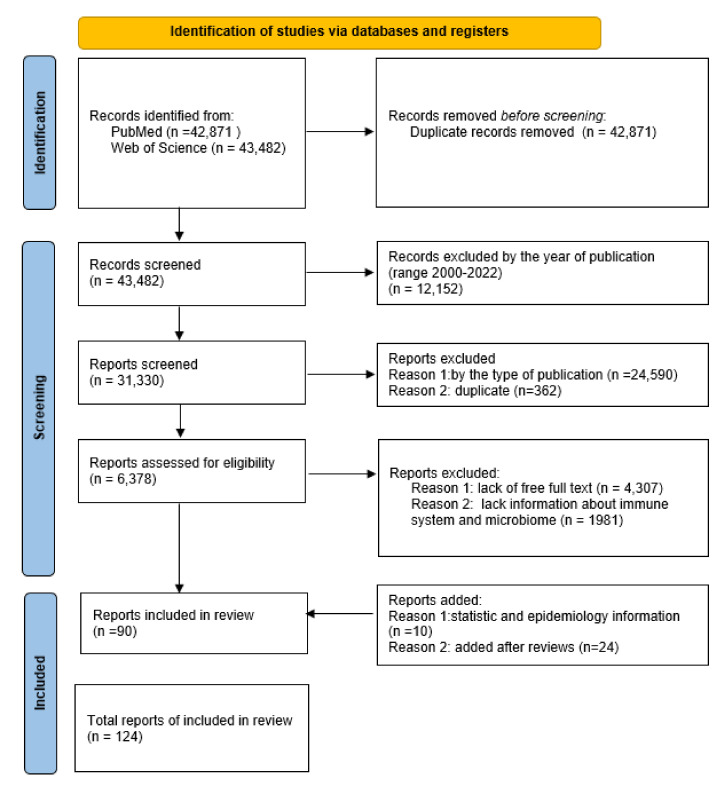
The flow diagram of the study’s inclusion and exclusion criteria.

The analysis allowing for the exploration of the discussed topic was carried out on the basis of information gathered from two databases: PubMed and Web of Science. Initial identification of the articles was based on a keyword search for “endometrial cancer”, “Endometrium”, and “Cancer”. Duplicate entries from both databases were rejected. Then, the list of publications was narrowed down by using appropriate filters. The first was the year the article was published, using the 2000–2022 range. The second criterion was the type of publication, which included clinical trials, reviews, and systematic reviews (all duplicate items were rejected). Then, for further analysis, only articles of which the full text was available, and containing such keywords as “microbiota”, “microbiome”, “immune system”, or “immune response”, were selected. After filtering the data, there were 90 articles that met the above criteria. The possibility of including each work for publication was thoroughly assessed. Additionally, 34 works on statistical and epidemiological information were included, as well as issues indicated by reviewers. Ultimately, 124 articles were included in the review (Figure 2).

## 2. Endometrial and Uterine Microbiota

### 2.1. Physiological Role of Endometrial and Uterine Microbiota

The specific interaction between the upper female reproductive tract’s microbiota, along with their communication, is still not fully understood. The vaginal microbiota plays a critical role in the prevention of a variety of urogenital disorders, including bacterial vaginosis, fungal infections, sexually transmitted infections, urinary tract infections, and HIV [30]. The current thinking links this to the presence of lactic-acid-producing bacteria—primarily *Lactobacillus* sp.—in the vagina, which are assumed to serve critical protective functions by contributing to the decreasing pH of the vaginal microenvironment through the generation of lactic acid, by creating a variety of bacteriostatic and bactericidal substances, or via competitive exclusion [31].

Since the advent of next-generation sequencing (NGS) technology, researchers have been able to examine the bacterial composition of the uterus on a far larger scale than was previously possible using culture-dependent approaches. The described method called metataxonomics (NGS—a sequencing technology to sort the DNA of entire bacterial genomes in form of 16S rRNAs) found that the probable molecular functions of the endometrial microorganisms are connected to cell metabolism, motility, genetic information, immunology, and signaling pathways [28,32].

### 2.2. Composition of Endometrial and Uterine Microbiota

So far, little research has been devoted to the nature of the endometrial microbiota, which is why this is a topic that still requires intensive research to explain the role of microorganisms in maintaining symbiosis, as well as the development of dysbiosis, causing infertility along with neoplastic changes [33]. Currently, the literature contains several works on the analysis of the microfloral composition of the female reproductive system and its periodic changes resulting from hormonal changes or pregnancy [34]. The endometrium is a special tissue that lines the female reproductive organs, and shows a series of periodic changes, such as rapid proliferation, secretory transformation, angiogenesis, interstitial edema, or desquamation [35]. As shown in the studies available, not merely the composition of the microbiota, but also the composition of immunocompetent cells and the pattern of expression of inflammatory genes undergo intense changes depending on the changing phases of the endometrium [36,37].

Studies by Mitchell et al. showed that *Lactobacillus* is the leading type of microorganism found in the uterine and vaginal endometrium, demonstrating that the presence of low levels of bacteria in the uterus—characterized by the presence of *Lactobacillus* sp., followed by *Gardnerella* sp., *Atopobium* sp., *Prevotella* sp. and *Sneathia* sp.—was not associated with significant inflammatory response [29]. Similarly, Fang et al. confirmed the vaginal and endometrial microbiome compositions of patients in various conditions, including healthy women, patients with endometrial polyps, and patients with chronic endometritis, proving significant differences between the vaginal and the endometrial microbiome, in which *Lactobacillus* sp., *Gardnerella* sp., *Bifidobacterium* sp., *Alteromonas* sp., and *Streptococcus* sp. were considerably more prevalent in healthy endometrial tissue [38]. Likewise, Moreno et al. also determined that the endometrium is primarily populated by *Lactobacillus* sp., followed by *Gardnerella* sp., *Bifidobacterium* sp., *Streptococcus* sp., and *Prevotella* sp. [39]. A study carried out by Moreno et al. in 2022 showed that the presence of bacteria of the genus *Lactobacillus* was negatively correlated with the occurrence of pathogenic bacteria, and positively correlated with commensal bacteria, which may be of significant importance for the stability of the endometrial ecosystem. It was also shown that the loss of *Lactobacillus* sp. and the presence of specific pathogenic bacteria—such as *Atopobium* sp., *Bifidobacterium* sp., *Chryseobacterium* sp., *Gardnerella* sp., *Streptococcus* sp., or *Klebsiella* sp. in endometrial fluid, and/or *Bifidobacterium* sp., *Gardnerella* sp., *Klebsiella* sp., and *Neisseria* sp. in endometrial biopsies—were additionally associated with non-successful reproductive results [40]. The study also indicated that the dysbiotic endometrial microflora profile consisting of *Atopobium* sp., *Bifidobacterium* sp., *Chryseobacterium* sp., *Gardnerella* sp., *Haemophilus* sp., *Klebsiella* sp., *Neisseria* sp., *Staphylococcus* sp., and *Streptococcus* sp. was associated with unsuccessful pregnancies and low live-birth rates. In contrast, *Lactobacillus* was consistently enriched in patients with high live-birth scores [40]. Additionally, the research conducted by Libby et al. in 2008 found that microorganisms such as *Atopobium vaginae* and *Gardnerella vaginalis* are the main bacterial pathogens associated with vaginitis, and are responsible for stimulating the innate immune response of vaginal epithelial cells [41]. Furthermore, to support this theory, it was shown that vaginal epithelial cells secrete IL-6 and IL-8 in response to *Atopobium vaginae* and *Gardnerella vaginalis*, but not to *Lactobacillus crispatus*. Additionally, the authors found that *Atopobium vaginae* induced elevated levels of IL-6, IL-8, and β-defensin 4 antimicrobial peptide transcripts. In contrast, the response of vaginal epithelial cells to microorganisms was mediated by the Toll-like receptor 2 (TLR 2), which required the MyD88 adapter protein and involved activation of the NF-κB signaling pathway. The results presented by the researchers indicate that *Atopobium vaginae* and *Gardnerella vaginalis* stimulate the innate immune response of vaginal epithelial cells, leading to the localized production of cytokines and defensin, which may contribute to the pathogenesis of bacterial vaginosis [41].

**Figure 3:**
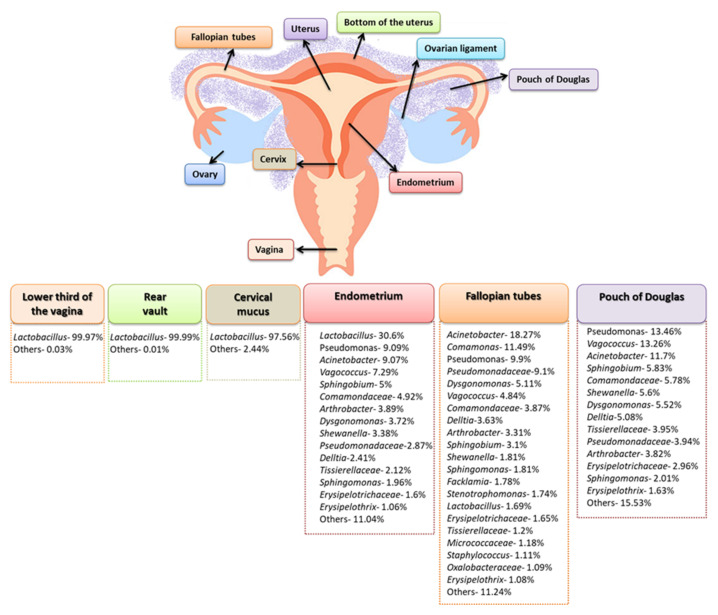
Differentiation in the composition of the female genital tract’s microbiota (based on [28]).

The literature also includes studies in which the composition of the microbiota of the female genital tract was analyzed in the context of individual places within, for example, the lower third of the vagina, the posterior vault, the left or right fallopian tubes, the endometrium, and cervical mucus collected from the cervical canal. Such a comprehensive approach resulted in an in-depth analysis of the composition of the microbiota, which turned out to be much more diverse. Chen et al. in 2017, showed that in the samples from the vagina and cervical mucus, the dominant type of bacteria was *Lactobacillus* sp., constituting 97.56–99.99% of the detected microorganisms. On the other hand, the tested samples from the peritoneal and uterine areas (which were collected during laparoscopy or laparotomy) showed a much greater variety. The microbiota composition of the endometrium, fallopian tubes, and peritoneal fluid were analyzed, showing a reduction in the amount of *Lactobacillus* sp. to 30.6% for the endometrium, and to 1.69% for the fallopian tubes. In addition, the percentage of *Pseudomonas* sp., *Acinetobacter* sp., and *Vagococcus* sp. species in the analyzed samples also changed (Figure 3) [28].

There are also published studies that do not support the abovementioned conclusions. A study by Verstraelen et al. in 2016 showed that the most abundant bacteria in the endometrium were of the genera *Bacteroides* (*B. xylanisolvens, B. thetaiotaomicron*, and *B. fragilis*) and *Pelomonas* [42]. Lu et al. in 2021, also showed that *Lactobacillus* is not the main species in the endometrium, proving a higher incidence of genera such as *Rhodococcus, Phyllobacterium, Sphingomonas, Bacteroides*, and *Bifidobacterium* [43]. Research by Winters et al. in 2019, who used 16S rRNA gene sequencing to investigate the presence of microflora in the reproductive systems of 25 women who had undergone a total hysterectomy for uterine fibroids or endometrial hyperplasia, found that *Acinetobacter* sp., *Pseudomonas* sp., *Comamonadaceae*, and *Cloacibacterium* sp. dominated endometrial bacterial profiles instead of *Lactobacillus* sp. [42].

### 2.3. Factors Influencing the Diversity of the Composition of the Endometrial and Uterine Microbiota

So, why are the results obtained by scientists so varied? As most uterine sampling is performed through the cervix, it is difficult to avoid cross-contamination of the cervical and vaginal microflora. In addition, uterine manipulators and cervical dilators may contribute to cervical cross-contamination, but these tools are rarely reported in studies. Nevertheless, strict adherence to pollution control measures and full descriptions of clinical processes are key elements allowing for reproducibility and, thus, better comparability of the obtained results in future studies [44,45].

**Figure 4:**
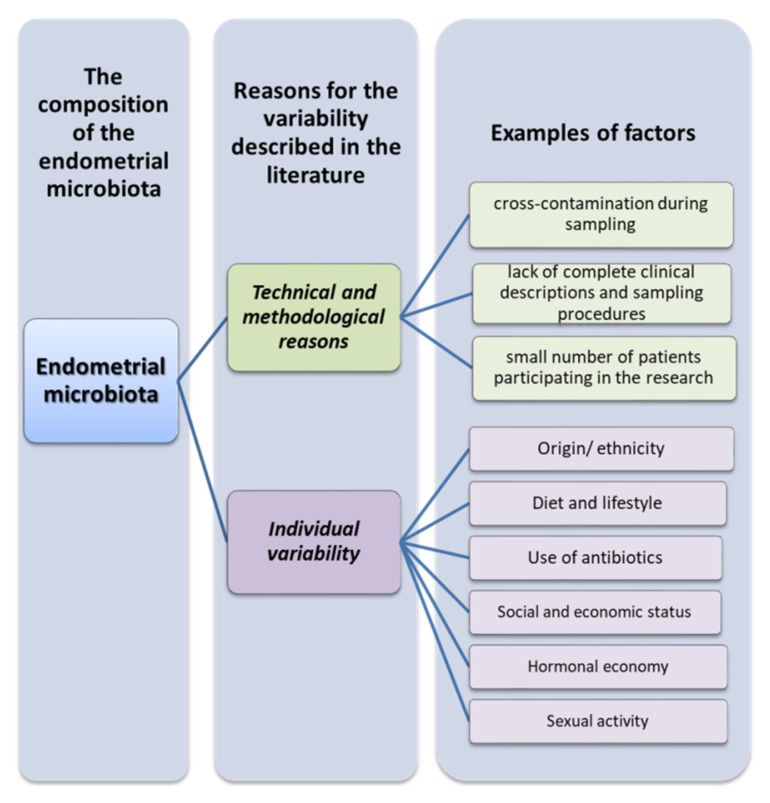
Reasons for the variation in the composition of the endometrial microbiota (based on [44,45,48,49]).

Another significant limitation in this area is the size of the cohort studies, due in part to difficulties in recruiting patients, and the technical difficulties involved in obtaining uterine specimens. Hysterectomy studies have shown that the rate of intrauterine bacterial colonization ranges from 0% in a cohort of 10 women in Finland [46] to 31% in 100 women in England [47]. Transcervical sampling studies show higher levels of intrauterine bacterial colonization, but the degree of cervical or vaginal contamination of the endometrial samples is unknown. Another important factor is the origin or ethnicity of the recruited patients. The genetic and phylogenetic composition of the human microbiome community varies considerably depending on the person, geographic location, first contact of infants with the environment, diet, lifestyle, or the use of antibiotics (Figure 3) [48]. Due to the generally small sample size and phylogenetic diversity, the statistical power of the results is reduced. If there are differences in the uterine microbiome between ethnic groups, this may have implications of risk factors for gynecological and reproductive consequences, but ought to also take into account changes in socioeconomic status and the environment, such as estrogen levels, puberty, menstruation, sexual activity, and nutrition (Figure 4) [49].

## 3. Composition of the Endometrial and Uterine Microbiota in Pathological Conditions, with Particular Emphasis on Endometrial Cancer

In line with the recent trend of microorganisms in the development and progression of various neoplastic diseases, scientists also decided to take a closer look at whether changes in the uterine microbiota and endometrium can significantly affect the development of cancer [50,51,52]. The limited data available in the literature show that women suffering from gynecological pathologies such as chronic endometritis [53], dysfunctional endometrial bleeding [54], endometriosis [55], endometrial polyps [38], endometrial cancer or hyperplasia [44,56], or infertility [57] have a changed composition of the uterine microbiota in relation to healthy women. All of these findings contribute to an increasing flow of information indicating that microbial diversity in the uterus is both physiologically and pathophysiologically important. Some observational studies have confirmed that female genital infections such as endometritis and pelvic inflammation are associated with the development of uterine cancer [58,59]. It is known that endometrial cancer is caused by an excess of estrogens but, theoretically, the colonization and infection of microorganisms may lead to chronic inflammation and, thus, contribute to an increased risk of uterine cancer [59]. However, at present, only a few studies address this extremely interesting and clinically important aspect.

In a 2016 study, Walther-Antonio et al. showed significant differences in the composition of microflora in the upper and lower segments of the female genitalia in women after hysterectomy due to endometrial cancer, endometrial hyperplasia, or benign conditions of the uterus. *Shigella* sp. and *Barnesiella* sp. have been identified as the most dominant microorganisms in women with endometritis or abnormal endometrial bleeding [60]. In addition, studies have shown the existence of significant differences in the composition of the endometrial microflora in hyperplasia compared to mild presentation, suggesting a role of the microflora in the early stages of cellular transformation, considering that hyperplasia is a precancerous stage of the endometrium [60]. Studies on sequencing the 16S rDNA V3-V5 region in samples from endometrial cancer patients showed that *Staphylococcus* sp., *Blautia* sp., and *Parabacteroides* sp. were particularly important in the benign cohort, while bacteria of the genera *Bacteroides* and *Faecalibacterium* were more important in the endometrial cancer cohort. This enabled confirmation that bacteria of the genus *Bacteroides* are the dominant taxa of the uterus. Additionally, the authors found that, in the lower genital tract (i.e., the vagina and cervix), the dominant taxa were *Prevotella* sp. and *Lactobacillus* sp., with *Stenotrophomonas* sp. and *Shigella* sp. being more characteristic in the benign cohort, and *Porphyromonas* being more common in patients diagnosed with endometrial cancer [60]. As shown by other studies available in the literature, *Porphyromonas* sp. have previously been isolated intracellularly, and it is possible that they are capable of interfering with normal cellular regulatory processes and, thus, triggering the process of carcinogenesis [60,61,62].

Similar results were reported by Walsh et al. in 2019, whose study aimed to determine the influence of patients’ characteristics (i.e., menopausal status, body mass index, and vaginal pH) and how these factors can lead to changes in the microbiome in patients with endometrial cancer [56]. The researchers found that the previously mentioned risk factors for endometrial cancer increase the diversity of the endometrial microbiota. Some samples showed *Anaerococcus tetradius*, *Anaerococcus lactolyticus*, *Peptoniphilus coxii*, and *Campylobacter ureolyticus*, which were correlated with the status of menopause. The presence of *Porphyromonas somerae*, which was confirmed by targeted quantitative polymerase chain reaction (qPCR), was identified as the most predictive microbial marker of endometrial cancer, and was not significantly correlated with the status of menopause. Further studies showed that the uterine microbiota containing *Porphyromonas somerae* was highly predictive of concomitant uterine cancer, with 86% positive predictive value when considering the status of menopause and obesity. As a result of both studies, *Porphyromonas somerae* is recognized as a biomarker of the disease [56].

Gonzalez-Bosquet et al. in 2021, compared the microbiome of the female upper genital tract, in the environment of endometrial and ovarian cancer, with control samples (fallopian tubes) [63]. They measured the differences in bacterial, archaea, and viral transcripts (BAVT) between high-grade serum carcinoma (HGSC) and endometrial carcinoma (EEC), and found 93 BAVTs different in expression between the two types of gynecological tumors, of which 7 significant differences were noted: *Salinibacter ruber*, *Bacillus tropicus*, *Pusillimonas* sp. ye3, *Riemerella anatipestifer*, *Nostocales cyanobacterium* HT-58-2, *Orgyia pseudotsugata,* and *Corynebacterium pseudotuberculosis* [63]. Nevertheless, in the controls, these BAVT species were the most abundant, while in the EEC samples—and even more so in the HGSC samples—BAVT expression was lower. These results may lead us to discover that BAVT expression is derived from genomic material embedded in the human genome that is associated with infectious and neoplastic signaling pathways [63].

Lu et al. in 2021, proved that in endometrial cancer the variability of the local microflora is reduced [43]. However, the endometrial cancer group had an increased number of *Micrococcus* sp. compared to the benign uterine lesions (BUL) group, while other genera (*Pseudoriibacter*, *Eubacterium*, *Rhodobacter*, *Vogesella*, *Bilophila*, *Rheinheimera*, and *Megamonas*) were enriched in the BUL group. The authors emphasized that the increase in the number of *Micrococcus* sp. in endometrial lesions may play a putative role in the development of endometrial cancer [43]. Moreover, the team of Li et al. in 2021, showed that the advanced severity of the disease is associated with a reduction in the diversity of the endometrial microbiome [62]. After completing their transcriptome research, they found altered expression of genes associated with fibrin breakdown in endometrial tumor tissues. Further correlation analysis showed that these genes were associated with an increase in the abundance of *Prevotella* sp., indicating that *Prevotella* may play a key role in mediating host fibrin breakdown leading to EC [62].

## 4. Microbial Dysbiosis and Its Immune Implications Related to Endometrial Cancer

There is a complex system in the human endometrium that prevents the risk of infection and allows the blastocyst to be implanted for fetal growth. Due to this fact, many scientists have suggested that the endometrium may function as a tertiary lymphoid organ, playing a key role in the immune surveillance of the uterus [64,65]. The results presented in the literature so far suggest that chronic inflammation caused by dysbiosis of the endometrial and uterine microbiome may be a risk factor for tumor development, and has potential as a new tumor biomarker and therapeutic target [43].

However, more than just the endometrial or uterine microbiome may be involved in the development of carcinogenesis within the female reproductive tract [66]. More and more research also concerns the role of the gut microbiota, which can also affect the health of the host through changes in its metabolome (i.e., the set of all metabolites present in the body, tissue, or cell) [67]. The gut microbiota also exerts a variety of effects on the gut environment, influencing the reproductive endocrine system by interacting with estrogens, androgens, insulin, and other hormones [68]. The gut microbiota is not only regulated by estrogens, but also plays a role in regulating the estrogen level itself. Estrogens are the main regulator of the gut microbiome, and the repertoire of genes in the gut microbiota capable of metabolizing estrogens is termed the “estrobolome” [69]. Studies have identified a cluster of genes that encode estrogen-metabolizing enzymes found only in bacteria in the gut microbiome [70,71,72]. Moreover, intestinal microorganisms play a crucial role in estrogen metabolism, as shown by the fact that the use of antibiotics reduces estrogen levels [73].

### 4.1. The Estrobolome and Endometrial Cancer

Estrogens play an important role in the development and maintenance of the female reproductive system [74]. They modulate the microenvironment of the lower female reproductive system through the pathways of increasing epithelial thickness, increasing glycogen concentration, increasing mucus secretion, promoting lactobacilli abundance and, indirectly, lactic acid production [75]. Endometrial cancer, endometriosis, and uterine fibroids are all proliferative disorders that have been associated with this hormonal dysfunction [76]. Increased levels of estrogens, as well as the lack of the opposing effect of progesterone, lead to inequalities between progesterone and estrogen production, influencing the epithelium, which may lead to uncontrolled profiling and hypertrophy, conducive to the development of endometrial cancer (especially type I) [77,78].

#### Estrogen Metabolism in the Intestines

**Figure 5:**
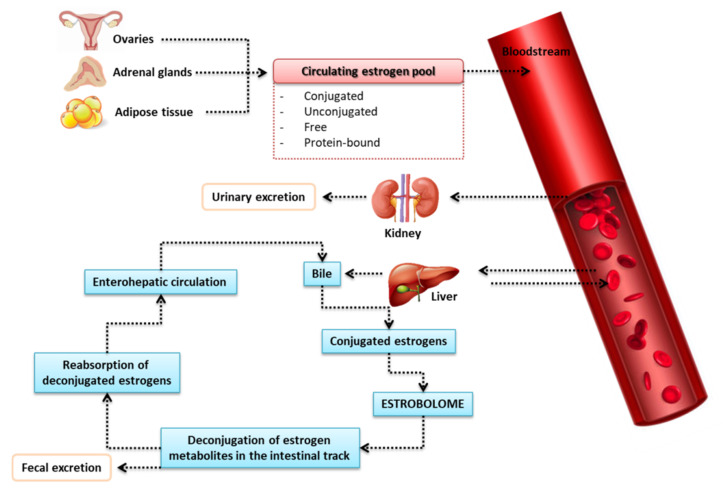
Metabolic changes of estrogen in the human body [69].

It is well known that the liver binds circulating estrogens (via glucuronidation) with glucuronic acid that do not bind to estrogen receptors. Glucuronic-acid-conjugated estrogens are more hydrophilic, allowing for biliary excretion (as bile salt forms), and are then released into the gut to remove conjugated toxins and hormones that are no longer required [79] (Figure 5). It has been proven that the intestinal microflora—especially changes in the intestinal microflora (dysbiosis)—is involved in the reactivation of estrogens via the bacterial enzymatic secretion of β-glucuronidase, detected in some intestinal bacteria, such as *Escherichia coli*, *Bacteroides fragilis*, and *Streptococcus agalactiae* [80], which are able to deconjugate glucuronic acid [66,81]. This results in reabsorption of active estrogen in the circulation and, thus, an increase in affinity for the target estrogen beta (ERβ) receptors [82,83]. Although in some studies the diversity of ERα was correlated with estradiol concentration, the mechanism remains unclear [82,84]. The activity of β-glucuronidase is crucial for the production of dangerous and carcinogenic metabolites in the gut, as well as for the reabsorption of various chemicals in the circulatory system, such as estrogens [85]. β-glucuronidase promotes estrogen receptor binding, and activation of these receptors promotes proliferation—an action well described in the pathogenesis of breast cancer—demonstrating the exchange between estrogen levels and the gut microbiota in hormone-dependent tumors such as breast cancer and endometrial cancer [66].

In one of their studies, Flores et al. found that non-ovarian estrogen levels were closely related to the amount and diversity of fecal microbiota in both postmenopausal women and men, with the taxa Clostridia within Firmicutes and three genera of the Ruminococcaceae family being the most related [82]. Moreover, the activity of -glucuronidase has been linked to the levels of estrone—but not estrogen—in urine. However, estrogen levels in pre-menopausal women are not affected by the microbiota or the activity of glucuronidase [82]. It should be noted that dysbiosis goes both ways, and can lead to a reduction in the diversity of the intestinal microflora that inhibits the activity of glucuronidase, resulting in less deconjugation of estrogens and phytoestrogens to their circulating and active forms. Decreased estrogen levels influence the activation of estrogen receptors, which may result in hyperestrogenic diseases such as obesity, metabolic syndrome, cardiovascular diseases, and cognitive reduction [69,86].

### 4.2. The Importance of Inflammation in the Immunopathogenesis of Endometrial Cancer

It is well known that there are many examples supporting the hypothesis of correlation between inflammation and cancer. A possible mechanism responsible for the link between the uterine microbiota and endometrial cancer is the association of bacterial toxins with tumor-promoting metabolites, which may lead to chronic bacterial inflammation with cytokine release by host cells [87]. A crucial role of inflammation in the initiation, promotion, malignant degeneration, invasion, and metastasis of various types of cancer has been established, but the importance of this process in the development and progression of endometrial cancer is still not fully understood, and requires further thorough research [88,89].

The first references to the role of inflammation in the pathogenesis of endometrial cancer come from a study by Modugno et al. in 2005 [90]. Scientists initially suggested that chronic inflammation was correlated with endometrial cell division, increasing the risk of replication errors and ineffective DNA repair. Their research established a link between the inflammatory environment in the endometrial tissue, the production of cytokines and growth factors, and the development and progression of endometrial cancer. Additionally, the researchers suggested that all recognized risk factors for endometrial neoplasms were directly or indirectly involved in modulating the inflammatory system—such as estrogen activity, which was associated with an increased pro-inflammatory response in the endometrium [90].

Another important study was Dossus et al.’s (2011) analysis of the relationship between tumor necrosis factor (TNF) and endometrial cancer in a case–control analysis using data from the European Prospective Investigation into Cancer and Nutrition (EPIC) [91]. The presented results showed that TNF was significantly capable of promoting endometrial proliferation, and was involved in the angiogenesis process of the analyzed cases [91]. In fact, TNF has been associated with established risk factors for endometrial cancer, including increased local estrogen production, increased development of insulin resistance, and type 2 diabetes. Although scientists cannot exclude the possibility that the presence of a tumor increases the circulating inflammatory mediators, the findings suggesting the function of TNF and its receptors were of particular interest in view of the prospects of treatment with TNF inhibitors [91]. In 2013, Dossus et al. investigated the correlation of multiple serum biomarkers with endometrial cancer, using pre-diagnostic blood samples. These biomarkers included hormones, growth factors, and cytokines. Immunological factors such as TNF-α, TNF receptors 1 and 2, IL-6, and C-reactive protein (CRP) have been identified as inflammatory variables associated with an increased risk of endometrial cancer in postmenopausal women [92]. As part of the conducted case–control study, an attempt was made to establish the relationship between CRP, IL-6, IL-1RA (interleukin 1 receptor antagonist), and the degree to which these variables are associated with the risk of obesity and endometrial cancer [93,94]. Blood samples from 305 people who were prospectively and accidentally diagnosed with endometrial cancer and 574 healthy controls were tested. In conclusion, increased cytokine levels were strongly associated with an increased risk of endometrial cancer, and their association was mainly related to the obesity level of the patients, indicating that cytokines act as aromatase inhibitors in adipose tissue [93]. The research results presented by Dossus et al. in numerous studies indicate that some markers of inflammation are strongly associated with endometrial cancer.

Similar conclusions were also drawn by Trabert et al. in 2017, who assessed blood levels of 64 biomarkers of inflammation in the blood in 284 cases of endometrial cancer and 284 control cases in a nested control analysis of 6297 cases, in a screening test for prostate, lung, colorectal, and ovarian cancer [95]. Endometrial cancer risk was highest among obese women with the highest levels of inflammation and multiple markers of inflammation, including adipokines, inflammatory cytokines, angiogenic factors, and acute-phase proteins. Importantly, vascular endothelial growth factor A (VEGF-A) has been identified as a risk factor independent of BMI [95]. Although the local inflammation promoting cancer development remains unclear, the production of cytokine growth factors via dysbiosis—such as tumor necrosis factor—is uncertain. Local tissue and infiltrating inflammatory cells seem to play a key role [87]. To support this claim, a nationwide, retrospective, population-based cohort study found that pelvic inflammatory disease increased the risk of developing endometrial cancer 1.79-fold [59]. Chronic inflammation stimulates angiogenesis, cell proliferation, and the formation of free radicals that damage DNA and aid in the initiation and progression of cancer [92].

From the available data in the literature, it can be concluded that the microbiome may play a role in the first stage of inflammation, causing immunopathological changes that ultimately lead to the development of cancer [96,97]. Activation of the immune receptor triggers a cellular response through the inclusion of the nuclear factor kappa b (NF-kB), mitogen-activated, protein kinase (MAPK), or PI3K/AKT signaling pathways. When these signaling pathways are activated, pro-inflammatory cytokines (e.g., TNF, IL-6, and IL-8) and/or antimicrobial peptides that contribute to the development of the inflammatory response are produced [43]. Another important aspect is the influence of microRNAs (e.g., miRNAs, miRs) on the development and pathogenesis of endometrial cancer. MiRNAs are naturally occurring, short (21–23 nucleotides long), non-coding RNA fragments that have the biological ability to induce gene silencing. This means that miRNAs inhibit the expression of mRNAs encoding many proteins, by inhibiting translation or causing mRNA degradation [98]. In the pathogenesis of cancer, miRNAs are responsible for the dysregulation of a number of important cellular processes, such as proliferation, apoptosis, aging, and cell differentiation, which contribute to oncogenesis and metastasis [99]. According to the data in the literature, several miRNAs show specificity for certain types or kinds of tumor, including endometrial cancer [100]. Previous studies have shown that in the course of endometrial cancer an altered expression pattern of several miRNA molecules is observed, such as miR-34 [101], miR-99a, miR-100 [102], miR-101 [103], miR-106b [104], miR-125 [105], miR-130b [106], miR-194 [107], miR-199b [102].

The miR-34 molecule—and especially its miR-34b subtype—is strongly associated with the proliferation and invasion of endometrial cancer cells, while the miR-34a subtype inhibits proliferation, migration, and invasion by targeting Notch1 (Notch receptor 1) in cells of this type of tumor [101,108]. The study of Tores et al. demonstrated that the levels of miR-99a, miR-100, and miR-199b were increased in the sera of patients with endometrioid cancer [102]. Other studies conducted by many scientists have shown that overexpression of miR-125b can function as an oncogene, while lowering the expression of miRNAs such as miR-106b, miR-194, and miR-130b can inhibit the growth of neoplastic cells in aggressive forms of endometrial cancer [104,105,106,107]. The activity of miR-101 still remains the subject of intensive research, due to the fact that it appears to be a two-way mechanism of action. This molecule has been shown to have suppressor activity in endometrial cancer cells (decreased tissue expression and inhibition of serum cell proliferation), and there is evidence that miR-101 may also act as an oncogenic molecule [109]. The research conducted by Konno et al. based on microarray techniques shows that EZH2 (histone methyltransferase inhibitors), MCL-1 (myeloid cell leukemia-1) and FOS (Fos proto-oncogene, AP-1 transcription factor subunit) are direct targets miR-101. Silencing these genes mimics the tumor-suppression effect observed in promoting the function of microRNA-101 [103]. As shown by research conducted by many scientists, the loss of suppressive miRNA activity significantly affects the process of oncogenesis and the progression of endometrial cancer [110]. Therefore, intensive research has been initiated to determine the targets of miRNAs in the pathogenesis of cancer diseases. One such target is the PI3K/AKT signaling pathway [111]. Genetic evidence in the literature shows that this pathway is a central mechanism that controls the characteristics of the epithelial–mesenchymal transition (EMT) in promoting cancer cell invasion, chemoresistance, and cancer stem cell (CSC) properties [112,113,114]. MiRNAs act as mediators, promoting and inhibiting the mechanism of the PI3K/AKT pathway in endometrial cancer. All of these findings indicate that miRNAs can be used as diagnostic markers in the course of endometrial cancer.

Further research into the underlying cause of chronic endometritis is very much needed to prevent, treat, and perhaps even cure endometrial cancer one day, while also providing a better understanding of the role of the microbiome in the long term.

### 4.3. Other Mechanisms Leading to Endometrial Dysbiosis and Endometrial Cancer Immunopathogenesis

In addition to inflammation and the secretion of cytokines by the host cells, the microbiota can promote cancer in many other ways, such as by preventing apoptosis, stimulating proliferation, and promoting genomic instability, all of which are hallmarks of cancer. Scientists have hypothesized that the uterine microflora may have a pathogenic effect on the endometrial epithelium. By regulating transcription factors and other genomic and epigenetic modifications, the presence of uterine microflora can influence the genomic stability of the uterine epithelium. Microbiota can promote carcinogenesis by releasing genotoxins that can damage the host’s DNA. This may directly induce cell carcinogenesis or autophagy [87]. Reducing expression at the junction between cells is a key way to break the epithelial barrier, allowing bacteria to pass between epithelial cells. Similarly, the breakdown of the extracellular matrix by matrix metalloproteinases violates the integrity of the epithelial barrier. Metabolites produced by microbes, such as short-chain fatty acids (SCFAs), can promote the growth of some bacteria while inhibiting the growth of others. The disease can also be caused by reactive oxygen species (ROS) and changes in the pH of the uterine microenvironment [50].

Inflammation caused by TLR activation and subsequent pro-inflammatory pathways can attract immune cells and result in the production of antimicrobial peptides (AMPs). Female genital tract epithelial and neutrophil cells are the main secretors of AMPs under the influence of inflammatory or bacterial stimuli. AMPs then exhibit a variety of activities, including bacterial opsonization and direct microbial death, resulting in lower bacterial abundance. So far, AMPs described in the female gynecological pathway include cathelicidins, defensins, lysozymes, whey acidic proteins, iron metabolism proteins, S100 proteins, type-C lectins, kinocidins, histones, BPI, TSP-1, lipophilin, cystatin A, ubiquitin, and phospholipase A2. TLR signaling may also influence the formation of membrane-bound and secreted mucins, which may impact colonization [96,115]. Since it is well known that the expression of pattern-recognition receptors such as Toll-like receptors (TLRs) increases with tumor growth, the next challenge will be to identify interactions that characterize microbial interactions in the uterus involving TLRs, so as to better understand progression of endometrial cancer [116,117].

Based on the collected data in the literature, scientists speculate that microbes interact with the endometrial epithelium and/or change the immunity of the mucosa through several mechanisms, including altered expression of genes encoding proteins involved in the inflammatory response, proliferation, and apoptosis in the endometrium, or through a series of immune-mediated changes, such as abnormal expression of leukocyte subsets, altered secretion of antibodies and cytokines, and immunoglobulins [118,119]. Consequently, these events—isolated or in combination—can ultimately disrupt various processes and promote the development of pathological conditions such as chronic endometritis, eventually resulting in the development of neoplastic diseases.

## 5. Conclusions and Research Prospects for the Future

To date, the structure of the endometrial hyperplasia microbiome can be distinguished from that of the benign uterine state, suggesting either a role of the microbiome in the early stages of cellular transformation or a well-known response to changes in the physiological or chemical gradient in the host cell. Additionally, the uterine microbiome can stimulate the production of pro-inflammatory cytokines in endometrial cells, including IL-1α, IL-1β, IL-17α, and TNF-α, which are involved in the carcinogenesis of various cancers. Due to the estrogen-dependent nature of endometrial cancer, dysbiosis of not only of the uterus, but also the intestinal microflora, is indirectly involved in carcinogenesis, causing an increase in serum estrogen levels by reactivating estrogen metabolites. Mechanical studies using in vitro and in vivo models, along with animal models, are necessary to determine the role of the microflora and specific bacterial species in initiating and/or maintaining cancer in the female reproductive system, including the uterus.

In the therapeutic setting, there is a significant interest and demand to diagnose and improve endometrial dysfunctions in order to treat uterine dysbiosis, despite the fact that there is no standardized technique for testing the endometrial microbial composition, nor for treating uterine dysbiosis. New therapies, such as probiotic and prebiotic supplements and microbiota transplants, are still poorly understood, and there are insufficient data to support the use of probiotics for enhancing and maintaining the appropriate microbiota composition. It is extremely challenging to verify the clinical usefulness of commercially available probiotics, due to the small sample size of the research and the variety in the strains of bacteria employed, duration of treatment, and patient lifestyle, all of which can alter the effects of probiotic use. Concerning the human uterus, a number of studies have examined the effect of probiotic supplements on the endometrial microbiota, but primarily in conjunction with antibiotic therapy [120,121,122,123]. Implementing alternative modulators for uterine microorganisms is a highly demanded and clinically relevant area of research.

Advances in macrobiotics and metagenomics have allowed researchers to begin identifying microbial communities and/or specific bacterial species that can cause pathological conditions in the female reproductive tract and, thus, contribute to the development and progression of cancer. Nevertheless, in the future, clinical trials with larger cohorts are required to validate and extend these initial findings to potential clinical use, as illustrated in the International Cancer Microbiome Consortium’s 2019 statement that long-term cohort data are required to support the role of the human microbiome as a critical driver of cancer’s etiopathogenesis. In addition, future epidemiological research must include women of different races, age groups, ethnicities, and socioeconomic status, as significant differences in the composition of the vaginal microflora have been identified between women of different ethnic origins. Additionally, it seems necessary to explain the influence of endometrial microbiome biodiversity on the reproductive process and the commensal or pathogenic relationships between microorganisms and endometrial cells. Furthermore, scientists should also look for answers to questions about the mechanisms of bacterial colonization and survival in the endometrial environment, and the actions of signaling pathways potentially activated by these microorganisms (with particular emphasis on the metabolites they synthesize). Only with an interdisciplinary and comprehensive approach to the issue of the role of the immune system and changes in the endometrial and uterine microbiota will it be possible to understand how their mutual correlation contributes to the development and progression of gynecological neoplasms.

## Data Availability

Not applicable.
